# P-1957. Spatiotemporal Association of COVID-19 Cases and Mortality with Exposure to Wildfire Particulate Matter in 2020

**DOI:** 10.1093/ofid/ofae631.2116

**Published:** 2025-01-29

**Authors:** Thomas C McHale, David R Boulware, Kelly Searle, Leda Kobziar, Phineas Lampman, Julio C Zuniga-Moya, Ben Papadopoulos, Andrej Spec, Naomi Hauser, George R Thompson

**Affiliations:** University of Minnesota, Atlanta, Georgia; University of Minnesota, Atlanta, Georgia; University of Minnesota, Atlanta, Georgia; University of Idaho, Coeur d'Alene, Idaho; University of Idaho, Coeur d'Alene, Idaho; Washington University School of Medicine in St. Louis, St. Louis, Missouri; Washington University of St. Louis, St. Louis, Missouri; Washington University in St. Louis, St. Louis, MO; University of California Davis, Sacramento, California; University of California Davis Medical Center, Sacramento, CA

## Abstract

**Background:**

Climate change is anticipated to have profound effects on human health including infectious diseases. Wildfires have increased in frequency and intensity due to changes in precipitation and have been linked to worsening respiratory infections. We aimed to demonstrate whether an association existed between wildfire smoke and COVID-19 in California during 2020.

**Figure:**
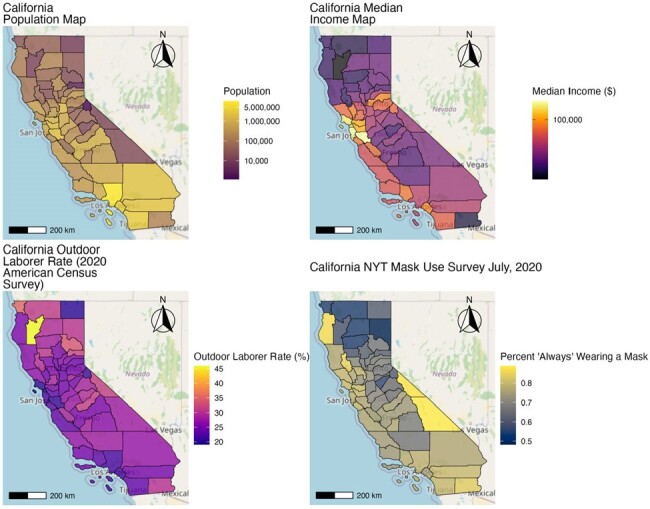

Demographic data by county in California based on the 2020 American Census Survey and New York Times mask-use survey conducted in July 2020. The population of California is generally higher in the southern region, especially Los Angeles County. The median income is highest in the San Francisco Bay area. The outdoor laborer rate is generally higher in northern California, especially the rural county of Trinity. Self-reported mask use in July 2020 was high throughout the state, but lowest in the northern region.

**Methods:**

We performed a population-based ecologic cohort study in California in 2020 using a spatial autoregressive model to test for associations between wildfire smoke and COVID-19 case and death incidence while adjusting for demographic and environmental factors. The counties of California were the units of analysis and all persons who lived in California were accounted for in the data. We used publicly available levels of particulate matter < 2.5 µm (µg/m^3^ PM_2.5_) specifically attributable to wildfire smoke in California during 2020.

**Figure:**
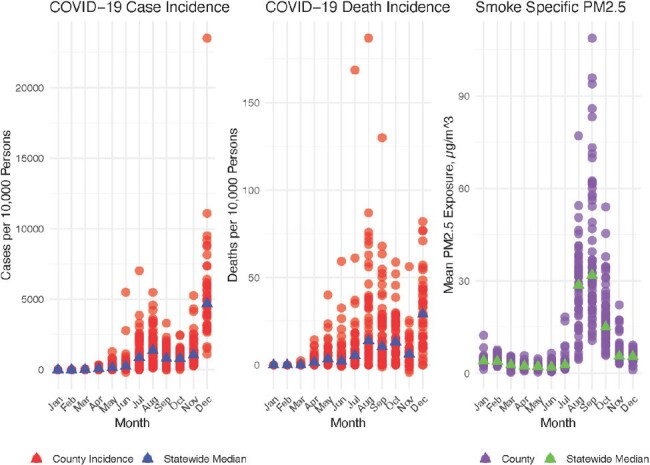

The median COVID-19 case and death incidence for each county in California and the overall case and death incidence in each county in California and the statewide county median case and death incidence is displayed in the first two plots below. The mean wildfire smoke PM2.5 exposure (µg/m3) in each California county and the median smoke exposure among California counties is displayed in the third plot. There was a surge in COVID-19 cases and deaths in California in the summer and winter of 2020 but a large degree of variation across counties. There was a surge in smoke exposure in the fall of 2020 and a large degree of variation across counties.

**Results:**

In a model adjusted for spatial autocorrelation, environmental and demographic confounders, we found as smoke exposure increased per each 10 µg/m^3^ in PM_2.5_, there was an average increase of 175 COVID-19 cases/10,000 persons the following month (1-month lag) at the county level (P< 0.001) and an increase of 1.86 COVID-19 deaths/10,000 persons the following month at the county level (P< 0.001). These findings were attenuated in the 2^nd^ month after smoke exposure where we found as smoke increased per each 10 µg/m^3^ in PM_2.5_ there was an average increase of 65.9 COVID-19 cases/10,000 persons the following 2^nd^ month but no increase in deaths.

**Figure:**
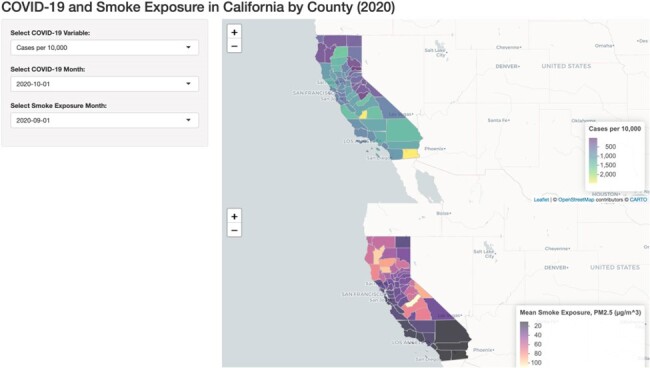

Screenshot of the interactive Shiny app displaying COVID-19 cases, deaths, and smoke PM2.5 for each county in California and each month of 2020. Link: https://mchal053.shinyapps.io/smoke-covid/.

**Conclusion:**

2020 was a particularly strong year for wildfires in California and a unique year for infectious diseases with the COVID-19 pandemic. Our findings, using a spatially adjusted autoregressive model which controlled for important confounding variables, demonstrated that wildfire smoke exposure likely increased COVID-19 acquisition potentially via epithelial injury. This may have implications for the transmission risk for other upper respiratory tract infections.

Table 1

**Figure:**
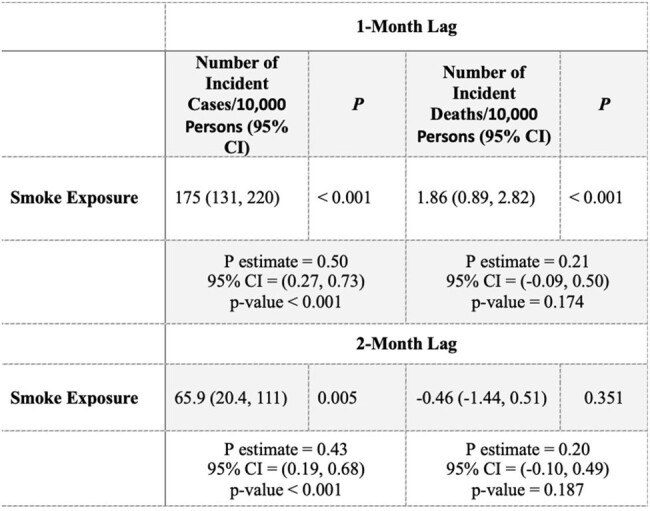

Adjusted spatial lag model for the COVID-19 case and death incidence per 10,000 persons per 10 µg/m3 increase in PM2.5 smoke exposure in 2020. The model is adjusted for median income, outdoor laborer rate, average 2020 temperature per county, monthly 2020 precipitation per county, average elevation, month of the year, and rate of respondents reporting “Always” wearing a mask when in public within 6-feet of other another person. We found very strong evidence for a 1-month lag increase of 175 COIVD-19 cases/10,000 persons and 1.86 deaths/10,000 persons and a 2-month lag increase of 65.9 COVID-19 cases/10,000 persons. There was no 2-month lag increase in COVID-19 mortality.

**Disclosures:**

Andrej Spec, MD, MSCI, F2G: Grant/Research Support George R. Thompson, III, MD, Astellas: Advisor/Consultant|Cidara: Advisor/Consultant|Cidara: Grant/Research Support|F2G: Advisor/Consultant|F2G: Grant/Research Support|Melinta: Advisor/Consultant|Melinta: Grant/Research Support|Mundipharma: Advisor/Consultant|Mundipharma: Grant/Research Support|Pfizer: Advisor/Consultant

